# Clinical records anonymisation and text extraction (CRATE): an open-source software system

**DOI:** 10.1186/s12911-017-0437-1

**Published:** 2017-04-26

**Authors:** Rudolf N. Cardinal

**Affiliations:** 1 Behavioural and Clinical Neuroscience Institute, Department of Psychiatry, University of Cambridge, Sir William Hardy Building, Downing Site, Cambridge, CB2 3EB UK; 2 Cambridgeshire & Peterborough NHS Foundation Trust and Cambridge University Hospitals NHS Foundation Trust, Liaison Psychiatry Service, Box 190, Cambridge Biomedical Campus, Cambridge, CB2 0QQ UK

**Keywords:** Anonymisation, De-identification, Clinical informatics, Electronic medical records, Open-source software, Pseudonymisation, Psychiatry

## Abstract

**Background:**

Electronic medical records contain information of value for research, but contain identifiable and often highly sensitive confidential information. Patient-identifiable information cannot in general be shared outside clinical care teams without explicit consent, but anonymisation/de-identification allows research uses of clinical data without explicit consent.

**Results:**

This article presents CRATE (Clinical Records Anonymisation and Text Extraction), an open-source software system with separable functions: (1) it anonymises or de-identifies arbitrary relational databases, with sensitivity and precision similar to previous comparable systems; (2) it uses public secure cryptographic methods to map patient identifiers to research identifiers (pseudonyms); (3) it connects relational databases to external tools for natural language processing; (4) it provides a web front end for research and administrative functions; and (5) it supports a specific model through which patients may consent to be contacted about research.

**Conclusions:**

Creation and management of a research database from sensitive clinical records with secure pseudonym generation, full-text indexing, and a consent-to-contact process is possible and practical using entirely free and open-source software.

## Background

Electronic medical records (EMRs) have considerable value for medical research. However, full EMRs identify patients completely and often contain highly sensitive confidential information. A key principle of information security is that patient-identifiable information should not be shared outside the clinical care-giving team without explicit consent, outside certain exceptional circumstances [1–3]. Anonymisation or de-identification allows researchers to be given access to material derived from identifiable clinical records, without explicit consent. This approach is endorsed and promised by the UK National Health Service (NHS) Constitution for England [4, 5] and has been used with success, including in psychiatry [6].

The terms “anonymisation” and “de-identification” are sometimes used interchangeably [7–9]; alternatively, “anonymisation” is used in a stronger sense to denote an irreversible form of de-identification [9]. De-identification carries several technical challenges [10]. In a simple structured database, such as a haematology database storing full blood count results, the bulk of de-identification may be accomplished simply by omitting key identifiers (such as names, dates of birth, and hospital numbers). However, in many databases, much valuable information may be in free text — notes, letters, and other documents — rather than in structured fields. This is a common feature of clinical records in psychiatry. The value in such data may be to a human reading the de-identified text directly, or through the creation of structured data from free text using natural language processing (NLP) software. De-identifying free text is more complex [6, 11, 12]. Finally, it is usually required that multiple records from the same patient are identified as being related in the de-identified database. This requires a mechanism for creating a patient-specific research identifier in a manner that does not violate the security of de-identification, and often in a way that patient identity can be looked up by administrators (so-called pseudonymised information [2]); these are problems of cryptography.

Existing tools for de-identification exist, using replacement rules and/or machine learning. However, some are not open-source [13], while many open-source tools are tailored to specific types of source record [12, 14], rather than embedding de-identification in a system for processing arbitrary relational databases. Large-scale EMRs often employ relational databases, so practical solutions to the problem of large-scale EMR de-identification need to manage the interface to such databases. Many de-identification systems do not address in full the cryptographic problem of pseudonym generation that accompanies de-identification in practice [13]. Some systems de-identify structured information in relational databases but do not de-identify free text [15]. Here I present an open-source system enabling the de-identification of arbitrary relational databases, with a particular focus on the application of de-identifying EMRs in psychiatry. To my knowledge, its novel contribution is the de-identification of both structured data and free text directly from one relational database to another, with pseudonym generation based on established cryptographic methods, in open-source software. In addition, it supports other aspects of the overall management of a secure research database, including the application of NLP tools directly to relational databases, researcher access, and a consent-for-contact process.

## Implementation

**Fig. 1:**
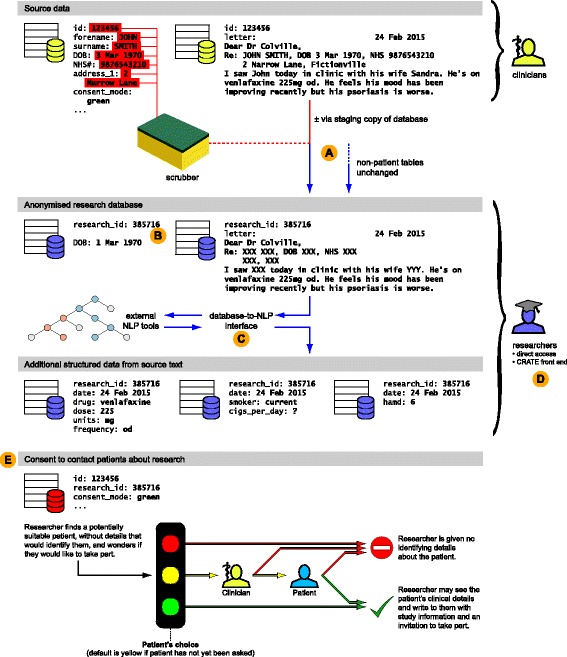
Overview of the roles that CRATE can play in the creation of a research database. The figure shows a schematic of a full EMR containing sensitive and identifiable information, its processing into a pseudonymised research database, and methods through which researchers may use the research database to contact patients about research, while preserving anonymity for those who have not consented to be contacted. Key functions of CRATE are shown, as follows. **a** Anonymisation of source data in a relational database framework, using identifiers in the source data to “scrub” free text. In this example the date of birth has also been partially obscured. **b** Generation of crypographically secure research IDs using hashed message authentication codes and one-time pads. An integer transient research ID is illustrated; full research IDs use longer hexadecimal digests. **c** Provision of a managed relational database interface to natural language processing tools such as GATE. **d** Provision of an optional web front end to a research database. **e** Management of a consent-to-contact process. The anonymisation, NLP, front end, and consent-to-contact components are modular and usable separately

CRATE (Clinical Records Anonymisation and Text Extraction) comprises tools for anonymising or de-identifying relational databases, a system for applying NLP tools to relational databases, a web front end for accessing the resulting research database, and a web front end for implementing a specific consent-for-contact model through which patients may be approached about research participation directly or via their clinicians (Fig. 1). It is intended as a toolchain covering all steps from a clinical relational database containing patient-identifiable data to a de-identified research relational database, with structured information from NLP tools added. In what follows, the term “source database” will be used to refer to the original EMR, or a copy thereof, which contains identifiable information, and “destination database” refers to the de-identified database created by CRATE.

The system aims to de-identify free text using information present in the source database, by suppressing direct identifiers and, optionally, reducing detail for indirect identifiers [8]. Typically, identifying information includes names, dates of birth, hospital numbers, address components, telephone numbers, e-mail addresses, aliases, and names of family members or other non-professional contacts [8].

The design follows that of the CRIS de-identifier [6, 13], but with improvements in the handling of database structure, reliance only on open-source technologies, and a different set of de-identification and pseudonym algorithms. Unlike CRIS, CRATE operates purely with relational databases, without an intervening XML step. CRATE can therefore perform patient-independent processing, such as copying system lookup tables (reducing data pre-processing requirements compared to CRIS), and handles database NULL values, which indicate the absence of a value, without modification (obviating the need to handle “dummy” values such as 1 January 1900 to indicate an absent date). CRATE itself is free and open-source software (FOSS) and relies only on FOSS, including for full-text indexing to support rapid searches for words in a corpus of text. It has extensible de-identification features, including automatic handling of typographical errors (described below). It uses published secure cryptographic methods for mapping patient identifiers (PIDs) to research pseudonyms (here termed research identifiers, RIDs), such that disclosure to approved users of individual PID/RID pairs does not compromise the security of the system as a whole. CRATE supports pseudonymisation, but the storage of pseudonyms in the destination database can be suppressed, if anonymisation (in the sense of irreversible removal of identifying material) is desired.

### Open-source software components

All components of the system are FOSS or rely on third-party FOSS tools. The CRATE system itself is written in Python [16] with some Java [17]. As a Python program it is cross-platform; it has been tested under 64-bit Ubuntu Linux 14.04 and higher [18, 19] and 64-bit Windows 10 [20] and Windows 2008 Server.

Database access for de-identification and NLP is supported via the SQLAlchemy library [21], which supports multiple database back-ends. CRATE has been tested with Microsoft SQL Server, MySQL, and PostgreSQL. NLP is supported via GATE [22], MedEx-UIMA [23], and Python regular expressions, and can be extended to other NLP frameworks.

The web site components of CRATE use an industry-standard FOSS stack. Under Linux, this includes the Django web framework [24], Gunicorn as the back-end web server [25], a front-end web server such as Apache [26] to provide secure hypertext transfer protocol access (HTTPS, via secure sockets layer, SSL), the supervisord task control program [27], and the Celery distributed task queue [28] using the RabbitMQ messaging system [29]. Under Windows, Gunicorn is replaced with CherryPy [30], which can also serve as a front-end web server providing HTTPS/SSL if required, and supervisord is replaced by a custom Windows service.

### Preliminary database processing

Only minimal data pre-processing is necessary. The computer(s) running CRATE should be able to “see” all databases containing the source data, locally or over a fast secure network. The source database should be a copy of the original clinical database with read-only access, not the “live” copy, so that its contents do not change during the anonymisation/de-identification process. Every table containing patient-identifiable information must have a column containing the database-wide patient identification number (PID), such as a local institutional identifier. If this is not true of the original clinical database, views should be created to add this information to existing tables.

The host institution may choose to add additional information to the source database(s) prior to de-identification. However, linking of multiple clinical databases prior to de-identification is unnecessary so long as those databases all contain a suitable common identifier (such as an NHS number), as these identifiers can be encoded in a way that allows several de-identified databases to be linked subsequently, via methods described below.

The software proceeds according to the instructions in a data dictionary, a file in tab-separated value (TSV) format containing one row for each column in the source database(s). Data dictionaries may be automatically generated from a source database, following a configurable process, but must be subject to human inspection before use, and normally require some customization.

### Anonymisation/de-identification methods

Relational databases are composed of tables having columns (fields) and rows (records). The CRATE system is designed to copy and de-identify arbitrary relational databases, with the caveat that any table requiring de-identification must have a column containing a database-wide patient identification number (PID). If the source database does not meet that constraint, a pseudo-column containing the PID must be added (for example, by creating views on existing tables). Tables not requiring anonymisation/de-identification, such as system lookup tables that do not contain patient-specific information, may be copied unchanged.

The data dictionary defines column names and structured query language (SQL) data types for the source and destination databases, and permits the renaming of columns in the process. It also defines special flags to control de-identification, as follows. Fields can be marked as containing sensitive information suitable for scrubbing with, so-called *scrub-source* fields. An example would be a structured field containing a patient’s forename, or another containing their date of birth. Alternatively, fields can be marked as text that may contain patient information and/or third-party information (relating to other people, such as the patient’s family, and usually excepting healthcare professionals [13]), so-called *sensitive free text* fields. As de-identification proceeds, the sensitive text is replaced (masked) by a customisable value, such as “[___]” for patient information and “[…]” for third-party information. The administrator may choose which information to scrub with, according to their specific de-identification requirements [8] — for example, information about a patient’s school might be weakly or strongly identifying, depending on the number of other patients sharing such a characteristic [8], might be important or unimportant for specific research reasons, and preservation or removal of such information might be required under local ethics and information governance rules, or applicable national regulations [8, 31].

Scrubbing is performed by regular expressions (regexes) [32]. For any individual scrub-source field, the software creates a custom regular expression for scrubbing, according to the administrator’s instructions regarding the type of information stored in that field. The following broad methods are defined. (All examples below are fictional and any resemblance to real persons is entirely coincidental.)

- *Scrub as words.* A source string (text from a scrub-source field in the source database) is split into chunks using all non-alphanumeric characters as boundaries, and each chunk is used for scrubbing. Thus, a scrub-source field containing “John Al’Rahem” will scrub all instances of “John”, “Al”, or “Rahem” from all sensitive free-text fields relating to that patient.
  - ◦ Safe (whitelisted) words may be configured, to omit them from scrubbing (typically, short words such as “a”, “of”, “the”, or “street” that may appear in patient initials or addresses but do not have to be excised from text).
  - ◦ Default suffixes may be applied for scrubbing, such as the suffix “s”, permitting de-identification if “Robert’s” is misspelled “Roberts”.
  - ◦ For more conservative scrubbing, up to a certain number of typographical errors (character insertions, deletions, or substitutions) may be permitted (using the fuzzy-matching feature of Python’s regex module [33]). With this method, if “Jakob” is misspelled “Jacob”, the error will still be scrubbed.
  - ◦ A minimum word length may be set for typographical errors to apply. The default value is 4. Without this option, for example, a patient name of “Ian” might remove all instances of “in” — an inconvenience, but also a clue to the name if the word “in” could be inferred from surrounding text.
  - ◦ A minimum string length may be set for scrubbing as well. If this is at its lowest and most conservative value of 1, then the address “4 Privet Drive” would scrub the “4” from phrases such “last episode 4–5 years ago” as well as from the address. The default value is 2.
- *Scrub as a phrase.* In this more restrictive text scrubbing method, the word sequence is only scrubbed if all words occur in order. Arbitrary non-alphanumeric separators are allowed between words. Whereas the “words” method is suited to names, the “phrase” method is better suited to addresses. It prevents, for example, “4 Privet Drive” from scrubbing dose information from “risperidone 4 mg/day”. The price is that it is less comprehensive at scrubbing slightly incorrect address information (e.g., “29 Acacia Avenue”, entered by mistake for “29 Acacia Road”, would be scrubbed to “[___] [___] Avenue” by the “words” method, but not scrubbed by the “phrase” method).
- *Scrub as a number.* A string or number from the source database is first converted into a digit string; for example “(01223) 123456” is converted to “01223123456”. This digit string is then used to scrub regardless of spacing or punctuation (thus a scrub-source field containing “123 456” will scrub the numbers from strings such as “M123456”, “NHS#123456”, “123 456”, “(123) 456”, or “123456”).
- *Scrub as an alphanumeric code.* This method operates as for numeric scrubbing, but allows any sequence of alphanumeric characters to be scrubbed, not just digits. This is effective for postcodes (thus a scrub-source field containing “CB12 3DE” will scrub “CB123DE”, “CB12-3DE”, and so on). No other semantic rules are applied.
- *Scrub as a date.* Dates may be expressed in very variable ways. The CRATE scrubber breaks dates into their component parts of day, month, and year, allows a wide variety of equivalents for each, and will deal with a wide variety of formats. Thus a source date of 7 January 2013 can be used to scrub all of “07 Jan 2013”, “7 January 13”, “7/1/13”, “1/7/13”, “Jan 7 2013”, “2013/01/07”, “2013-01-07”, “7th January 13”, “Jan 7th 13”, “07.01.13”, “7.1.2013”, “20130107T0123”, “20130107”, or many other variations. Though dates are scrubbed in a variety of formats, they are treated as simple points in time, to remove potential identifiers such as dates of birth, without using more sophisticated temporal information extraction methods [34]; this means that indirect or unusual references to dates (such as “he was born on Easter Day in 1950”) may be missed.

A significant weakness of this system is that the software can only scrub information that is present in the source database.

In addition, some categories of *non-specific* scrubber can be configured. For example, the administrator can require that all numbers of specified lengths be removed. Specifying lengths of 10 and 11 would remove UK NHS numbers and modern UK telephone numbers in full format, respectively, whether or not these are specified in scrub-source fields. The potential price is, of course, the removal of relevant but non-identifiable information in a similar format. UK postcodes can also be scrubbed in non-specific fashion. Non-specifically scrubbed information is replaced by a third distinct place-holder, such as “[~~~]”. Words can be blacklisted (always removed) or whitelisted (never removed). Other than for blacklisted/whitelisted words, dictionary methods are not used.

Examples of these regular expressions are shown in Table 1. Scrubbers are applied in the general order: (1) nonspecific scrubbers, (2) patient scrubbers, and (3) third-party scrubbers. Within the latter two categories, individual scrubbers are applied in sequence in the order specified by the data dictionary, which is configured by the administrator.

**Table 1:** Example regular expressions

Method	Source information	Example of case-insensitive regular expression(s) for scrubbing
Words	John Al’Rahem	► \b**John**\b►\b**Rahem**\b
Phrase	4 Privet Drive	► \b**4**\W+**Privet**\W+**Drive**\b
Number	(01223) 123456	► (?< !\d)**0**\W*1\W*2\W*2\W*3\W*1\W*2\W*3\W*4\W*5\W*6(?!\d)
Alphanumeric code	CB12 3DE	► \b**C**\W*B\W*1\W*2\W*3\W*D\W*E\b
Date	31 Dec 2016	► 0*31(?:st|nd|rd|th)?\W*(?:0*12|**Dec**(?:**ember**)?)\W*(?:**20**)?**16** ► (?:0*12|**Dec**(?:**ember**)?)\W*0*31(?:st|nd|rd|th)?\W*(?:**20**)?**16** ► (?:**20**)?**16**\W*(?:0*12|**Dec**(?:**ember**)?)\W*0*31(?:st|nd|rd|th)?
Nonspecific: 10-digit numbers	–	► (?< !\d)[0-9][ \t]*[0-9][ \t]*[0-9][ \t]*[0-9][ \t]*[0-9][ \t]*[0-9][ \t]*[0-9][ \t]*[0-9][ \t]*[0-9][ \t]*[0-9](?!\d)
Nonspecific: UK postcodes	–	► \b[A-Z][0-9]\s*[0-9][A-Z][A-Z]\b ► \b[A-Z][0-9][0-9]\s*[0-9][A-Z][A-Z]\b ► \b[A-Z][A-Z][0-9]\s*[0-9][A-Z][A-Z]\b ► \b[A-Z][A-Z][0-9][0-9]\s*[0-9][A-Z][A-Z]\b ► \b[A-Z][0-9][A-Z]\s*[0-9][A-Z][A-Z]\b ► \b[A-Z][A-Z][0-9][A-Z]\s*[0-9][A-Z][A-Z]\b

For the method specified in the first column, the de-identifying software will take individual instances of sensitive data, from scrub-source fields (an example is shown in the second column), and generate regular expressions (third column) with which to scrub sensitive free-text fields. Examples are shown at their default settings; all methods can be configured further (e.g., to allow typographical errors, set minimum word lengths for scrubbing, or to change boundary detection conditions for the regular expressions), as described in the text. All source examples are fictional. Emphasis added for clarity

All categories of scrubber can be configured to operate at word boundaries only, or at any location within the source string. The latter is more conservative but can potentially scrub too much permissible text. All regex searches are case-insensitive.

Beyond the configuration options offered for the scrubbers, the system can be altered or extended freely as required by end users with programming experience, using Python to create appropriate regular expressions or other methods as desired.

One or more *alteration methods* can be specified for each field. These include:

- omission of the field from the destination (for unnecessary or sensitive information);
- scrubbing the field of sensitive information (typically for sensitive free-text source fields);
- partially obscuring dates to the first of the month (e.g., for patient date-of-birth fields, allowing calculation of approximate age without exact dates of birth [13]);
- treating the field contents as a filename or binary large object (BLOB) document and extracting plain text from the document (currently supported for DOC, DOCM, DOCX, HTML, DOT, ODT, PDF, RTF, XML, and plain text files), to assist in extracting free text from binary files attached to the EMR, where the EMR contains a reference to a file held elsewhere on disk or contains the document as a BLOB;
- removal of HTML tags or escape sequences;
- skipping of individual rows based on inclusion or exclusion values (e.g., to restrict to current versions of records whose full editing history is present in a table).

Indexes, including full-text indexes (if supported), may be added to the destination fields. Indexes greatly speed up database queries when applied correctly, and full-text indexes greatly speed up searching for keywords within long passages of text (e.g., [35]).

The overall process of anonymisation/de-identification (Fig. 1a) is therefore as follows:

- Non-patient tables are copied without scrubbing.
- A single non-specific scrubber is created.
- A column from a master table in the source database that contains all possible PIDs is used to generate each PID in turn.
- For each PID, the program works through all scrub-source fields for that patient, generating one agglomerated regex that describes all patient-identifiable data, and a second regex for all third-party data.
- For that patient, the program then works through all rows from all tables, applying the scrubbers to any sensitive free-text fields and applying any other alterations.

An ability to opt out is often an important part of overall information governance procedures. CRATE’s opt-out list allows specific patients to be omitted entirely, and wiped from the database if they were processed in a previous iteration. The opt-out list can be managed manually from an administrative database table, and/or from disk files, and/or from opt-out marker fields in the source database.

Debugging options allow the de-identification of small quantities of data to tune the data dictionary and de-identification parameters appropriately to the institution’s source data prior to a full run. A further tool allows a sample set of raw and de-identified documents to be extracted for human before-and-after comparison with a text comparison tool such as Meld [36].

### Multiple databases

Multiple databases can be copied and de-identified jointly, if they share a common PID. In this situation, information held in one database can be used to de-identify information held in the other database, and vice versa, by creating a common pool of sensitive information with which to “scrub”.

If multiple databases do not share a common PID, so that they must be de-identified separately, but nevertheless have an identifier in common (such as two partially overlapping databases having different local numbering systems but with NHS number information), that identifier can be treated as a “master PID” (MPID) and hashed consistently across the databases, allowing subsequent linkage, as described below.

### Translation from patient identifier to research identifier

The de-identified research database requires patient identifier equivalents: RIDs (Fig. 1b). These distinguish different patients, link tables in the research database, and if desired can serve as the basis for re-identification via a managed consent-to-contact process. Several properties should be satisfied by a good PID-to-RID mapping system. (1) It is vital that the mapping be *one-way*, so that researchers cannot generate the PID from the RID. (2) The algorithm must be *collision-resistant*, so that no two PIDs are mapped to the same RID. (3) It is desirable that the algorithm be *published*, so that the source code for the de-identifier contains no valuable algorithmic secrets, and so that users can be assured of its cryptographic basis. (4) It is highly desirable that *knowledge of individual PID/RID pairs* should not break the algorithm. For example, if a clinician recognizes one of their patients from the de-identified information (by virtue of their additional clinical knowledge), or is deliberately given a RID so that they may access additional analysed data present in the research database, then they will be aware of one or more PID/RID pairs. This should not allow them to calculate other PIDs from other RIDs. (5) The RID should be of a relatively convenient length and format for researchers to operate with. (6) It is desirable but not always necessary that the mapping be *reproducible*, so that if the research database is destroyed it can be regenerated without loss of consistency with previous RIDs, and so that two databases can be de-identified independently using a consistent PID-to-RID mapping and then linked on RID.

The simplest method that satisfies criteria 1–5 is a “one-time pad” method, such as choosing an unused random number for each new PID. However, this is not reproducible. A reproducible method requires a *secret key* that is combined with the PID to produce the RID.

Simple cryptographic hash functions using the secret key as a salt satisfy most of these criteria, but not necessarily criterion 4. Since PIDs are typically simple, such as an integer sequence (or in the case of NHS numbers, an integer sequence with a checksum digit), it is possible that knowledge of one PID/RID pair allows generation of another [37]. An appropriate choice of algorithm that also satisfies criterion 4 is a *hashed message authentication code* (HMAC) based on a cryptographically secure hash function [37].

The CRATE system converts PIDs to string-form hexadecimal digests of a hash. It allows use of HMAC-MD5, which produces a short (32-character) digest; MD5 is cryptographically flawed but HMAC-MD5 overcomes its weaknesses, such that known collision attacks on MD5 do not break HMAC-MD5 [37]. CRATE also offers HMAC-SHA-256 and HMAC-SHA-512, which produce 64- and 128-character digests respectively. SHA-256 and SHA-512 are currently secure in their own right [38].

The secret key is part of the institution’s configuration of CRATE, not of the software itself. CRATE allows separate keys to be specified for the PID (used for creating the RID) and the MPID (used for creating the master research identifier, MRID). The use of a consistent secret key allows multiple databases to be de-identified separately yet subsequently linked via the encrypted RID equivalents. The mapping is stored in a separate secret database, enabling reverse look-up.

Because 32- to 128-character digests can be inconvenient for simple queries, the system also generates a transient integer research ID (TRID), using a “one-time pad” system. TRIDs have a 1:1 mapping to RID, which is maintained across incremental database updates, but this 1:1 mapping is destroyed and regenerated when a full database rebuild is performed. Because an auto-incrementing integer might yield a predictable and therefore insecure relationship between PID and RID, the TRID is generated pseudorandomly (with a random or a pre-specified seed for the random number generator). The TRID, therefore, is convenient for within-query use (for efficient linkage of different tables within the research database), and for that reason is used as the primary key for tables containing patient-identifiable information, but researchers should use RIDs or MRIDs for any longer-term identification of records — for example, if information must be preserved across subsequent database rebuilds.

This method also allows for a variety of methods of data linkage across sites [10]. Suppose institution A and institution B have information that they wish to link, and that linkage is possible because of a shared identifier, such as an NHS number. Information governance and data protection rules within the NHS will typically prohibit A and B sharing identifiable information without explicit consent. A traditional method in the UK to permit research in this setting is for the institutions to apply for an exemption to this rule under Section 251 of the NHS Act 2006; to share and link the identifiable data (and often a small subset of it) prior to anonymisation/de-identification, either themselves or via a trusted third party C; and then to make the resulting jointly anonymised/de-identified data available to researchers with strict controls relating to access to the data. The use of appropriate cryptographic hashing allows, in principle, alternative methods. For example, A and B can hash their data independently, using a common method and shared secret salt, and then share some of the de-identified data (limiting the transport of identifiable material), which can be linked via the common hash. The salt used for this sharing does not have to be the same as that used by each institution for its routine internal work, and access to any stored mappings between different RIDs (generated using different salts) can be controlled as required. Salt secrecy is important: if identifiers such as NHS numbers are used as PIDs, it must be assumed that knowledge of the salt allows RID to PID lookup by brute-force hashing of all possible PIDs, since such PIDs are from a restricted range (an NHS number is a 9-digit number plus a tenth checksum digit, so there are fewer than 10^9^ possible NHS numbers).

### Incremental updates

Incremental updates can be much faster than full database runs, so CRATE supports incremental de-identification. The source database may change in several pertinent ways. New patients may be added to the source database; new information may be added for existing patients; information may be deleted; and aspects of confidential patient data may change (for example, a new alias may be added to the EMR for the patient by clinical or clinical administrative staff: William, known as Bill). The CRATE de-identifier will handle incremental updates for any table with a suitable primary key (PK) that can uniquely identify each row. Other tables are dropped from the destination and recreated.

During de-identification, a hash is created for each source row and stored in the destination database (unless the administrator guarantees that the contents will not change), and similarly a hash of each patient’s scrubber is kept in a secret database. Rows present in a destination table but absent from the corresponding source are deleted. For each patient, the scrubber is calculated; if the scrubber’s hash differs from the stored version, then confidential information has changed and all destination records for that patient are reworked in full. Finally, rows that have changed (as judged by hash) or are new are processed.

Incremental updates are also supported for data dictionary creation, though human intervention is required to check data dictionaries.

Several database engines support hot-swapping of databases (e.g., [39]). This allows a lengthy de-identification process to run without disturbing the active research database, followed by a brief swap to the new database.

### Natural language processing system

NLP is supported via GATE [22], a general-purpose NLP framework; MedEx-UIMA [23], a tool for identifying references to medication; and Python-based regular expressions. NLP is run as a separate step from de-identification. Administrators can configure CRATE by defining one or more NLP definitions. Each NLP definition specifies a set of source fields (such as free-text progress notes, or fields containing text extracted from clinical documents), an NLP processor, and a set of output tables. Text from the source fields is fed to the NLP processor, which produces zero or more results, and into the output tables, along with metadata such as details of the source table/record.

CRATE provides a mechanism to couple multiple NLP tools into a relational database pipeline, and offers a class framework for implementing regular-expression NLP applications in Python. As GATE and MedEx-UIMA are Java-based systems that run as standalone processes, CRATE provides a system to encapsulate them for use with relational databases (Fig. 1c): text from the source fields is fed by CRATE’s Python-based code, via a process pipe connection (with or without temporary files, depending on the NLP tool’s requirements), to CRATE code written in Java, and thence to the external application. Each CRATE process loads and repeatedly reuses a single Java instance, as there is substantial overhead associated with loading the Java system. The external application processes the text. For example, a default example GATE application detects people’s names and cities within free text, and this would return XML output marked with Person and Location tags. CRATE sends these to a destination database, adding cross-references to the source material. For example, it might send Person information to a table containing fields such as “firstname”, “surname”, and “gender”. The output from different NLP tools can be fed into different destination tables, which is typically necessary to manage structural inconsistencies between the output of different NLP tools, or desirable to compare the performance of two different NLP tools having the same aim.

The accuracy of NLP depends on the performance of the specific external application (e.g., [23, 40]).

Incremental updates are supported in the same manner as for de-identification. The CRATE system is extensible to other NLP frameworks.

### Researcher access to data and audit

The final database can be queried using raw SQL, allowing researchers to use a variety of standard SQL tools including statistical software, and full auditing.

CRATE also provides a web-based front end interface to assist with visualization of the research database structure and queries, with content highlighting and auditing (Fig. 1d). It incorporates a simple SQL query builder but also permits researchers to enter raw SQL (requiring a read-only connection to the database for security against malicious input).

### Database administration functions and consent-to-contact system

Further secure web-based interfaces are provided for privileged functions, including reverse lookup of PIDs from RIDs.

The system also provides a specific consent-to-contact system (NHS Research Ethics reference 12/EE/0407). In this system, patients may consent or refuse to be contacted by researchers. When researchers submit a contact request using a RID, this is either translated to an approval notification containing the patient’s details (if the patient has consented) or is electronically rejected (if the patient has refused). An additional route, which can be actively selected by patients but which also operates as the default when patients have not been asked about research preferences, is that the researcher’s request is transmitted by institutional secure e-mail to the patient’s clinician, who may pass the request on to the patient or veto it (Fig. 1e). This approach satisfies the general principle of “consent or anonymise”: if the patient has previously consented, their identity can be made available to approved researchers, but if not, the first approach about research is from a member of their clinical care team, and researchers can see only anonymised data.

The consent-to-contact system is separable from the de-identification and the NLP systems.

### Other technical, institutional security, and information governance considerations

CRATE provides core de-identification functions, but implementing a secure research database using sensitive clinical data requires a good deal more that CRATE does not attempt to address. Additional technical considerations include restricting access to the research database and web site to computers within the institution’s secure network (± virtual private network) only; requiring that connections from researchers to the database computer use only encrypted HTTPS/SSL, even within the institution, to prevent “wire sniffing”; appropriate securing of the computer against other forms of access (using firewalls and other aspects of operating system security); and physical security, power protection, and backup systems for the hosting computer(s). Institutional and cultural considerations include an appropriate information governance framework, including information governance training and suitable contractual and professional obligations upon users; an oversight framework; Caldicott Guardian approval within the NHS, and appropriate engagement with all stakeholders [6].

## Performance

### Speed

Processing is parallelized. For de-identification, non-patient tables containing an integer PK have their rows distributed across multiple processes. Non-patient tables without an integer PK are assigned wholesale to different processes, as is the work of indexing. Patient tables have their work distributed by integer PID across multiple processes. For NLP, work is parallelized by integer PK. The system is capable of parallel processing across a cluster as well as on a multi-core machine, though a single high-performance multi-core machine is typically easier to manage.

#### Methods

De-identification speed was tested using a synthetic database of 1.5 Gb containing 1000 fictional patients, 100 notes per patient, and 1000 random words per note. The test computer was an 8-core × 3.5 GHz Intel i7-3770 K processor running Linux with 16 Gb RAM and SSD storage. All databases ran under MySQL; full-text indexing was not enabled.

#### Results

De-identification was performed at 14.2 Mb/s. In single-process profiling on a smaller similar test database of 100 patients, 51% of CPU time was spent in the core regex substitution function and 27% in database query/commit calls. An incremental update on an unchanged database ran 3.3 times faster. For execution of GATE’s demonstration “people and places” text recognition application, text was processed at 0.18 Mb/s (with the GATE framework being the primary consumer of CPU time).

### Accuracy of de-identification

#### Methods

For de-identification, a *target* was defined as a potential identifier present in the source text; a *non-target* as any other word in the source text; a *positive* response as scrubbing the source word (masking it by replacing it with a character sequence such as “[___]”); and a *negative* response as not masking it. A *hit* (true positive) was defined as the correct masking of a target; a *miss* (false negative) as a failure to mask a target; a *false alarm* (false positive) as the unnecessary masking of a non-target; and a *correct rejection* (true negative) as non-masking of a non-target. *Sensitivity = recall = true positive rate* was therefore defined as *P*(hit|target) = *P*(masked|identifier), and *precision = positive predictive value* as *P*(hit|positive) = *P*(hit|hit or false alarm) = *P*(identifier|masked).

*Known* identifiers were defined as identifiers marked as such in the source data; and *unknown identifiers* as those identifiers present in free-text fields but not marked as identifiers in the source data (e.g., aliases or family member names not recorded in structured form in the source database, which the anonymiser does not know about and therefore cannot mask, or where records were misfiled against the wrong patient) [13]. *All* identifiers encompass both known and unknown identifiers.

Identifiers were defined very liberally (including, for example, a single initial). Defined this liberally, some identifiers are intrinsically hard to mask with perfect precision/recall (e.g., if someone’s initial is a word such as “A” or “I”). Where an identifier was at times relevant for removal and at other times not (e.g., “Peterborough” occurring as part of a patient’s address and therefore being masked from part of a clinic or general practitioner address), the context was used by a human to determine if removal was necessary, thus providing a strict criterion against which to evaluate the algorithm. Precision deviated from 100% because of overlap between identifying and non-identifying information. For known identifiers, recall deviated from 100% only when spelling errors exceed the configured threshold (an adjustable parameter). The difference in recall between known identifiers and all identifiers, obviously, reflects a lack of coding of identifiers within the source data.

For scoring, words were counted in the conventional fashion for source data, and where masking occurred, a single mask was considered to be a single word in the results.

##### Settings common to all conditions

Source material forming part of the Cambridgeshire and Peterborough NHS Foundation Trust (CPFT) Research Database (Research Ethics reference 12/EE/0407) was used. One clinical document was sampled from each of 100 distinct patients to evaluate performance (yielding 50,274 words). The identifiers available for scrubbing included: CPFT patient identification numbers; NHS numbers; names (including recorded aliases); dates of birth; addresses; e-mail addresses; telephone numbers; UK National Insurance numbers; and family members’/carers’ names, dates of birth, addresses, e-mail addresses, and telephone numbers. Not all of this information was available for all patients. De-identification was performed with the following settings: default string suffix “s”; up to 1 typographical error detected; typographical error detection applied to scrub-source strings of 4 characters or more; a minimum string length of 1 for scrub-source text strings; no non-specific scrubbing; scrubbing of codes, dates, and strings at word boundaries but scrubbing of numbers at any location within the source string (regardless of word boundaries). A short whitelist was used: am, an, as, at, bd, by, he, if, is, it, me, mg, od, of, on, or, re, so, to, us, we, her, him, tds, she, the, you, road, street. De-identification was then tested using an “untuned” configuration and then progressively refined, applying a total of three different configurations of the software to the same text corpus, in an attempt to improve precision by reducing the false-alarm rate.

##### Condition 1

In this condition, the most stringent, town and county information was considered identifying (despite this being unrealistic). No geographical locations were whitelisted. Addresses were anonymised using the “words” method.

##### Condition 2

In this more realistic condition, town and county information was considered non-identifying. The commonest parts of local institutional addresses were whitelisted (Cambridge, Peterborough, Huntingdon, Cambs, Cambridgeshire).

##### Condition 3

Settings were as for Condition 2, except (a) whitelist terms were added relating to further geographical areas containing service sites of CPFT or its partners (Fulbourn, Wisbech, Ely, Saffron Walden, Essex, Norwich, Norfolk) or non-identifying towns causing frequent false alarms (March, a town that often appears in dates), and (b) addresses were anonymised as phrases rather than words.

#### Results

Results are shown in Table 2. Tuning the software to be aware of relevant local geographic locations reduced the false-alarm rate (from Condition 1 through to Condition 3). The software detected and masked all identifiers present in the source database, except for one miss in Condition 1 that was due to an address being mis-spelled with 2 typographical errors (1 above the software’s threshold). The results illustrate the principal weakness of this de-identification method: that, when considering all identifiers, misses occured because the identifying information was not recorded in structured form in the source database. Such “unknown” identifiers were primarily nicknames, and names of friends or family members. Misses were occasionally due to a document being misfiled in the wrong patient’s record. (The decrease in all-identifier sensitivity seen in Condition 3 was due to partially incorrect addresses, not present in the source, but matched in part using subset information in the previous conditions; e.g., “29 Acacia Road” marked as an identifier but entered in error as “29 Acacia Avenue” in the free text.)

**Table 2:** De-identification performance

Metric	Condition 1	Condition 2	Condition 3
Number of words in source text (*n*)	50,274	50,274	50,274
Hits	1,392	1,326	1,116
False alarms	275	132	25
*For known identifiers (those recorded as structured information in the source database):*			
Misses	1	0	0
Correct rejections	48,606	48,816	49,113
Sensitivity = recall	0.999	1	1
Precision	0.835	0.909	0.978
*For all identifiers (including those not recorded as structured information in the source database):*			
Misses	127	125	128
Correct rejections	48,480	48,691	49,005
Sensitivity = recall	0.916	0.914	0.897
Precision	0.835	0.909	0.978

Performance of the de-identifier on the same corpus of clinical documents, with three different specimen configurations. The conditions differed in the definition of “identifying information” used, in whitelisting of geographical location, and in the method used for detecting fragments of addresses (see text; these differences lead also to variation in the number of hits counted, for example whether successful masking of an address such as “29 Acacia Avenue” was counted as one hit, if masked to “[___]”, or several hits, if masked to “[___] [___] [___]”). A miss was defined as any identifier appearing in the destination text and identifiers were defined very liberally, including a single initial, so appearance of a single identifier in the destination text does not equate to identifying the patient concerned [13]

## Conclusions

This article presents an open-source cross-platform package capable of de-identification of generic relational databases, using public cryptographic methods for mapping patient identifiers to research IDs. The system is suitable for source materials containing substantial quantities of sensitive free text, such as electronic clinical records in psychiatry.

The de-identification results, particularly from the tuned state (sensitivity 1.0 and precision 0.978 for identifiers recorded in structured form in the source database, and sensitivity 0.897 and precision 0.978 for all identifiers), are comparable to specimen performance for the original CRIS de-identifier (sensitivity/recall 0.976 and precision 0.988 in one sample data set, and sensitivity 0.885 and precision 1.000 in another) [13]. Exact figures will depend on the data set used. Importantly, an organization can choose its own balance of sensitivity and precision for its own ends through appropriate configuration. A significant weakness of the system is that it relies on the presence of identifying information in the source database to de-identify free text. The use of an open-source system and public cryptographic algorithms allows others to manipulate the software freely, enabling other de-identification techniques [11, 12] to be brought into the system if required.

## Abbreviations

CPFT: Cambridgeshire and Peterborough NHS Foundation Trust
CRATE: Clinical Records Anonymisation and Text Extraction
CRIS: Clinical Record (formerly Case Register) Interactive Search
DOC: Microsoft Word 97–2003 document format
DOCM: Office Open XML document format with macros
DOCX: Office Open XML document format
DOT: Microsoft Word 97–2003 template format
EMR: Electronic medical record
FOSS: Free and open-source software
GATE: General Architecture for Text Engineering
HMAC: Hashed message authentication code
HTML: Hypertext markup language
HTTPS: Secure hypertext transfer protocol
LGPL: GNU Library/Lesser General Public License
MD5: Message Digest-5 algorithm
MIST: MITRE Identification Scrubber Toolkit
MPID: Master patient identifier
MRID: Master research identifier
NHS: UK National Health Service
NLP: Natural language processing
ODT: Open Document Format for text documents
PDF: Portable Document Format
PID: Patient identifier
PK: Primary key
RAM: Random access memory
RID: Research identifier
RTF: Rich Text Format
SHA: Secure Hash Algorithm
SQL: Structured Query Language
SSD: Solid-state drive
SSL: Secure sockets layer
TRID: Transient research identifier
TSV: Tab-separated value
UK: United Kingdom
XML: Extensible Markup Language
